# Bacteriophage infection and killing of intracellular *Mycobacterium abscessus*

**DOI:** 10.1128/mbio.02924-23

**Published:** 2023-12-07

**Authors:** Alan A. Schmalstig, Andrew Wiggins, Debbie Badillo, Katherine S. Wetzel, Graham F. Hatfull, Miriam Braunstein

**Affiliations:** 1 Department of Microbiology and Immunology, UNC School of Medicine, University of North Carolina at Chapel Hill, Chapel Hill, North Carolina, USA; 2 Department of Biological Sciences, University of Pittsburgh, Pittsburgh, Pennsylvania, USA; New York University School of Medicine, New York, New York, USA

**Keywords:** bacteriophage therapy, *Mycobacterium abscessus*, macrophages, intracellular bacteria, nontuberculous mycobacteria

## Abstract

*Mycobacterium abscessus* is a nontuberculous mycobacteria that contributes to the decline and death of patients with lung diseases such as cystic fibrosis and other muco-obstructive airway diseases. *M. abscessus* is challenging to treat due to its extensive antibiotic resistance and ability to survive inside mammalian cells. An alternative to antibiotics is the therapeutic use of bacteriophages (phages). There are recent cases of phage therapy being used to treat *M. abscessus* infections in people under compassionate-use conditions. However, little is known about the ability of phages to kill bacteria, such as *M. abscessus*, which reside in an intracellular environment. Here, we used *M. abscessus* strains and phages from recent phage therapy cases to determine if phages can enter mammalian cells and if they can infect and kill intracellular *M. abscessus*. Using fluorescence microscopy, we demonstrate phage uptake by macrophages and lung epithelial cells, and we further demonstrate phage infection of intracellular *M. abscessus* with fluorescent reporter phages. Transmission electron microscopy was additionally used to image phage infection of intracellular *M. abscessus*. Together, these findings provide the first visualizations of phage-*M. abscessus* interactions in an intracellular environment. Finally, we show that phage treatment can significantly reduce the intracellular burden of *M. abscessus* in a manner that depends on both the specific phage and mammalian cell type involved. These results demonstrate the potential to use phage therapy to treat intracellular bacteria, specifically *M. abscessus*, while also highlighting the importance of prescreening phage therapy candidates for activity in an intracellular environment.

**IMPORTANCE:**

As we rapidly approach a post-antibiotic era, bacteriophage (phage) therapy may offer a solution for treating drug-resistant bacteria. *Mycobacterium abscessus* is an emerging, multidrug-resistant pathogen that causes disease in people with cystic fibrosis, chronic obstructive pulmonary disease, and other underlying lung diseases. *M. abscessus* can survive inside host cells, a niche that can limit access to antibiotics. As current treatment options for *M. abscessus* infections often fail, there is an urgent need for alternative therapies. Phage therapy is being used to treat *M. abscessus* infections as an option of last resort. However, little is known about the ability of phages to kill bacteria in the host environment and specifically in an intracellular environment. Here, we demonstrate the ability of phages to enter mammalian cells and to infect and kill intracellular *M. abscessus*. These findings support the use of phages to treat intracellular bacterial pathogens.

## INTRODUCTION

The prevalence of antibiotic-resistant infections is on the rise. At the same time, development of new antibiotics is in decline (1–5). As a result, there are a growing number of bacterial infections that are in danger of becoming completely untreatable. Bacteriophage (phage) therapy is an alternative to antibiotics for treating bacterial infections. Lytic phages are viruses that infect and kill bacteria. The lytic phage lifecycle involves phage adsorption to a bacterial host, injection of genetic material, replication inside the bacterium, and lysis of the bacterium (6). The final lytic event releases new phage progeny killing the bacterium in the process. The potential of using phages as therapies for bacterial infections was discovered over 100 years ago (7). However, after the advent of antibiotics, phage therapy was largely forgotten by the West (8). In response to increasing antibiotic resistance, phage therapy is being reconsidered as an antibacterial treatment. Phages are employed in several recent compassionate-use cases to treat highly drug-resistant bacterial infections including *Acinetobacter baumannii*, *Pseudomonas aeruginosa*, and *Klebsiella pneumoniae* infections (9–11). Clinical trials are also starting to be performed (NCT05453578 and NCT04684641). Importantly, evidence indicates that phage administration is well tolerated and safe (12, 13). An advantage of phage therapy compared to antibiotics is the highly specific nature of phages for their bacterial host, which limits indiscriminate killing of commensal bacteria and off-target effects.

*Mycobacterium abscessus* (*M. abscessus*), a nontuberculous mycobacteria (NTM), is a highly drug-resistant bacterial pathogen in need of effective therapies. *M. abscessus* cases are increasing worldwide, and they are a particular concern for people with compromised immune systems and those with muco-obstructive lung diseases such as cystic fibrosis (CF), chronic obstructive pulmonary disease, and non-CF bronchiectasis (14–18). Due to its inherent and acquired antibiotic resistance, *M. abscessus* is extremely difficult to treat (19–23). Treatment involves multiple drugs, is lengthy, is associated with toxicity, and is often not curative (24, 25). One of the challenges of treating *M. abscessus* is that the bacterium has both extracellular and intracellular lifestyles that need to be targeted by therapy (26, 27). In particular, the ability of *M. abscessus* to survive in phagosomes inside macrophages is critical to the pathogenesis of this bacteria (20, 28, 29). Thus, when developing new therapies for *M. abscessus*, it is important to consider efficacy in the intracellular environment.

The unmet need for therapies to treat NTM infections has led to the use of phage therapy in 20 compassionate-use cases for whom all available treatment options had failed, primarily infections of *M. abscessus* (30–34). These cases employ lytic mycobacteriophages that are first determined to infect *M. abscessus* isolates of the patient (35). In one case, a CF patient with chronic untreatable *M. abscessus* infection was treated twice daily with a phage cocktail (31). Phage treatment was associated with improved lung pathology and conversion of sputum cultures to negative for *M. abscessus*. This improvement allowed the patient to get a lung transplant. Most impressively, the explanted lungs of the patient had no detectable *M. abscessus* suggesting complete eradication of the bacteria (31). However, favorable clinical or microbiological outcomes were only observed for 11 of the 15 cases where outcomes could be evaluated (30). Thus, a better understanding of the ability of phages to function as therapeutics and the challenges facing this approach is needed to improve phage therapy as a treatment for *M. abscessus*.

For intracellular bacteria, like *M. abscessus*, phage particles will need to reach and kill bacteria residing in mammalian cells. However, little is known about the ability of phages to infect bacteria in intracellular environments. Moreover, it is often assumed that phage therapy will only work with extracellular bacteria and not intracellular bacteria (36, 37). This assumption is based on the premise that phages will not be internalized by mammalian cells due to the lack of phage receptors on these cells. Additionally, for intracellular bacteria that reside in phagocytic cells, like macrophages, the possibility of immune responses clearing phages from the intracellular environment is often raised as a concern (38–40). However, among the relatively small number of studies of phage interactions with mammalian cells, there are reports of phages being taken up by phagocytic and epithelial cells (41, 42) and reports of phage killing intracellular bacteria in mammalian cells (43–46).

In this study, we explored the potential for phages to infect and kill intracellular *M. abscessus* in different mammalian cell types, using *M. abscessus* strains and phages from clinical cases. Our results demonstrate the ability of phages to enter mammalian cells and infect intracellular *M. abscessus*. Most importantly, we show that phages can kill intracellular *M. abscessus* in a phage- and mammalian cell type-dependent manner. These results provide a foundation for understanding and optimizing the use of phages as therapies for intracellular pathogens.

## MATERIALS AND METHODS

### Bacterial strains and growth conditions

*M. smegmatis* mc^2^155, *M. abscessus* subsp. *abscessus* (GD20), *M. abscessus* subsp. *massiliense* (GD82), and *M. abscessus* subsp. *abscessus* ATCC 19977 Δ*mbtH* (PM3492, gift from Martin Pavelka) strains were used (30, 32, 47, 48). *mbtH* is a glycopeptidolipid biosynthesis gene, and its deletion causes a rough colony morphology (47). This strain will be referred to as ATCC 19977 Rough. *M. smegmatis* and *M. abscessus* were cultured in Middlebrook 7H9 medium or on 7H10 agar with 1× albumin dextrose saline, 0.5% glycerol, and kanamycin 75 µg/mL as needed. Media were additionally supplemented with 0.05% Tween 80 for *M. smegmatis* or 0.1% tyloxapol for *M. abscessus*.

### Phage stocks and high-titer lysate preparation

BPsΔ*33*HTH-HRM10 (BPsΔ), ZoeJΔ45 (ZoeJΔ), Muddy, BPsΔ*32-33*_HRM10 mCherry (BPsΔ mCherry), and ZoeJΔ*43-45* mCherry (ZoeJΔ mCherry) were used in this study (49, 50). ZoeJ∆ mCherry was engineered using BRED (51); genes *43 (int*), *44*, and *45 (rep*) of ZoeJ (coordinates 33972-36489) were replaced with the mCherry expression cassette that was also used to make BPsΔ mCherry (49). Phage high-titer lysates were prepared on lawns of *M. smegmatis*, concentrated with polyethylene glycol precipitation, and purified with ultrafiltration (see supplemental methods).

### Bacterial strain construction

*M. abscessus* strains were transformed with pJH9.1 (green fluorescent protein [GFP]) (52) or pMSP12::mCherry (Addgene plasmid #30169) plasmids via electroporation as described in the supplemental methods.

### Phage quantitation by plaque assay

Tenfold dilutions of phage were spotted on 7H10 plates (without Tween 80) with top agar overlays containing *M. smegmatis*, *M. abscessus* GD20, or *M. abscessus* GD82. After 3–6 days of incubation at 37°C, plaque-forming units (PFU) were enumerated.

### Tissue culture

THP-1 cells, A549 cells, and murine bone marrow-derived macrophages (BMDMs) were grown and seeded for infection as described in the supplemental methods.

### *M. abscessus* infection of mammalian cells

Mammalian cells were seeded in eight-well chambered slides (Nunc Lab-Tek II, Thermo Fisher Scientific) at 1 × 10^6^ cells/mL (THP-1, BMDM) or 1.25 × 10^5^ cells/mL (A549). *M. abscessus* strains were grown to log phase, washed with phosphate-buffered saline (PBS) with 0.05% Tween 80 two times, and syringe passaged 10 times through a 27G needle. The dispersed *M. abscessus* culture was added to cell monolayers at a multiplicity of infection (MOI) of 10. After 3 h of infection, monolayers were washed with PBS three times. The final wash was replenished with RPMI or DMEM media containing 50 µg/mL amikacin (Sigma-Aldrich) to prevent growth of extracellular *M. abscessus*. At specific time points, mammalian cells were washed three times with PBS, lysed with 0.1% Triton X-100, and plated on 7H10 agar to enumerate intracellular *M. abscessus*.

### Fluorescent staining of phage

Phage were stained with SYBR Gold nucleic acid stain (Thermo Fisher Scientific) for 1 h at 4°C with a final concentration of 2.5×. Excess stain was removed by washing phage with 40 mL phage buffer (10 mM Tris pH 7.5, 10 mM MgSO_4_, 68 mM NaCl, 1 mM CaCl_2_) in an ultrafiltration centrifugation tube with a 100-kDa molecular weight cutoff (MWCO) (Pierce Protein Concentrator, Thermo Fisher Scientific).

### Fluorescence microscopy of phage uptake by mammalian cells

Cells were seeded at 1 × 10^6^ cells/mL (THP-1, BMDM) and 6.25 × 10^4^ cells/mL (A549) in eight-well chambered coverglass. SYBR Gold-stained phage were diluted in culture media and added at an MOI of 10^3^ or 10^5^ phage:mammalian cells. After 24 h, cells were washed three times with PBS to remove extracellular phage. Cells were then stained, as described in the supplemental methods, with CellMask deep red plasma membrane stain (Thermo Fisher Scientific), fixed with 4% paraformaldehyde, washed, and stained with 100 ng/mL diamidino-2-phenylindole (DAPI). Slides were imaged with an Olympus IX81 widefield microscope with a 40×/1.3 oil DIC UPlanFLN objective using Metamorph software version 7.10.2.240 and a Hamamatsu ORCA-Flash4.0 C13440 camera. Z-stacks were taken at 0.2-µm intervals, and AutoQuant software version X3.1.3 was used for 3D deconvolution of the images. Imaris Viewer software version 10.0.0 (Oxford Instruments) was used to confirm that phages were intracellular. FIJI software version 2.9.0/1.53t was used for color correction and image analysis (53). A maximum intensity Z projection was used to display all Z stacks. The percentage of macrophages with intracellular phage was counted per field of view from at least two independent experiments each with a minimum of 14 fields of view and a minimum of 300 mammalian cells counted.

### Fluorescence microscopy of phage infection of intracellular *M. abscessus*

Cells were seeded at 1 × 10^6^ cells/mL (THP-1, BMDM) and 1.25 × 10^5^ cells/mL (A549) in eight-well chambered coverglass. Mammalian cells were first infected with GFP-expressing *M. abscessus* at an MOI of 10 for 3 h, as described above. After three washes with PBS to remove extracellular bacteria, fresh media containing mCherry encoding reporter phage at an MOI of 10^4^ and 50 µg/mL amikacin were added to the *M. abscessus*-infected monolayer (49). After 24 h, the infected monolayers were washed three times with PBS. Cells were stained and imaged as described above with a 60×/1.42 oil DIC PlanApo objective. Image analysis was performed as described above. GFP and mCherry expressing bacteria were counted per field of view. Data were collected from two independent experiments each with a minimum of 12 fields of view and a minimum of 350 *M*. *abscessus* cells (GFP) counted per experiment.

### Transmission electron microscopy (TEM)

THP-1 and A549 cells were seeded in six-well tissue culture-treated plates (Corning) at 1 × 10^6^ and 2.5 × 10^4^ cell/mL, respectively. Mammalian cells were first infected with *M. abscessus* at an MOI of 10 for 3 h. Following three washes to remove extracellular bacteria, fresh media containing phage at an MOI of 10^4^ and 50 µg/mL amikacin were added to the *M. abscessus*-infected monolayer. After 24 or 48 h, the infected monolayers were washed three times with PBS, followed by a 3-minute incubation with phage inactivation buffer (43) (PIB; 40 mM citric acid, 10 mM KCl, 135 mM NaCl, pH 3.0), and three additional washes with PBS. Cells were then fixed in 2% paraformaldehyde/2.5% glutaraldehyde in 0.15 M sodium phosphate buffer, pH 7.4, for 1 h at room temperature and stored at 4°C. Cells were further processed and stained with uranyl acetate, followed by Reynolds’ lead citrate (54), as described in the supplemental methods. Samples were observed with a JEOL JEM-1230 transmission electron microscope operating at 80 kV (JEOL USA, Peabody, MA), and digital images were acquired using a Gatan Orius SC1000 CCD camera and Gatan Microscopy Suite 3.0 software (Gatan, Inc., Pleasanton, CA).

### Quantitation of intracellular *M. abscessus* following phage treatment

Mammalian cells were infected with *M. abscessus* at an MOI of 10 for 3 h, as described above. Following three washes to remove extracellular bacteria, fresh media containing phage at an MOI of 10^3^ or 10^5^ with 50 µg/mL amikacin were added to the monolayer. After 48 h, the cells were washed three times with PBS. To prevent free phage in lysate from infecting bacteria during outgrowth on agar, mammalian cells were lysed in PIB containing 0.1% Triton X-100 for 10 minutes and plated on 7H10 agar.

### Statistical analysis

All experiments were independently performed at least twice, and data are shown as the mean ± standard deviation. One-way analysis of variance (ANOVA) with Tukey’s post hoc test was performed for experiments quantitating *M. abscessus* colony-forming units (CFU) in mammalian cells (see Fig. 2 and 7). Kruskal–Wallis one-way ANOVA with Dunn’s post hoc test was performed for phage uptake by mammalian cells experiments (see Fig. 4). Mann–Whitney test was performed for mCherry reporter phage infection experiments (see Fig. 5). Statistical analyses were performed using GraphPad Prism software version 9.5.0 (San Diego, California USA).

## RESULTS

### Phage sensitivity of *M. abscessus* clinical isolates GD82 and GD20

**Fig 1:**
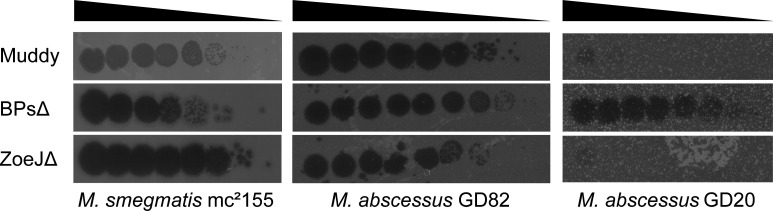
Phage sensitivity of specific mycobacterial strains. Serially diluted phages were spotted on lawns of *M. smegmatis* mc^2^155, *M. abscessus* GD82, or *M. abscessus* GD20. Cleared spots in the lawn indicate phage-mediated killing.

Two clinical isolates, *M. abscessus* subsp. *massiliense* GD82 and *M. abscessus* subsp. *abscessus* GD20, were chosen for this study (55). Both strains have rough colony morphotypes and were isolated from patients treated with phages in compassionate-use cases (30, 32). GD82 is from a bronchiectasis patient, and GD20 is from a CF patient (30, 32). These *M. abscessus* isolates were screened for susceptibility to three clinically relevant phages with siphoviral virion morphologies: BPsΔ*33*HTH-HRM10 (BPsΔ), ZoeJΔ45 (ZoeJΔ), and Muddy. BPsΔ and ZoeJΔ are lytic variants of the temperate parents BPs and ZoeJ. BPsΔ, ZoeJΔ, and Muddy each map to different genomic groups: Clusters G1, K2, and AB, respectively. The majority of *M. abscessus* phage therapy cases employ one or more of these phages. The GD82-infected patient was treated with BPsΔ, ZoeJΔ, and Muddy, while the GD20-infected patient was treated with BPsΔ and Itos phage (30, 32). Along with GD82 and GD20, we evaluated the ability of BPsΔ, ZoeJΔ, and Muddy to lyse *M. smegmatis* mc^2^155 (Fig. 1). *M. smegmatis* mc^2^155 and *M. abscessus* GD82 are sensitive to all three phages tested. *M. abscessus* GD20 is sensitive to BPsΔ but resistant to ZoeJΔ and Muddy.

### GD82 and GD20 grow in macrophages and lung epithelial cells

**Fig 2:**
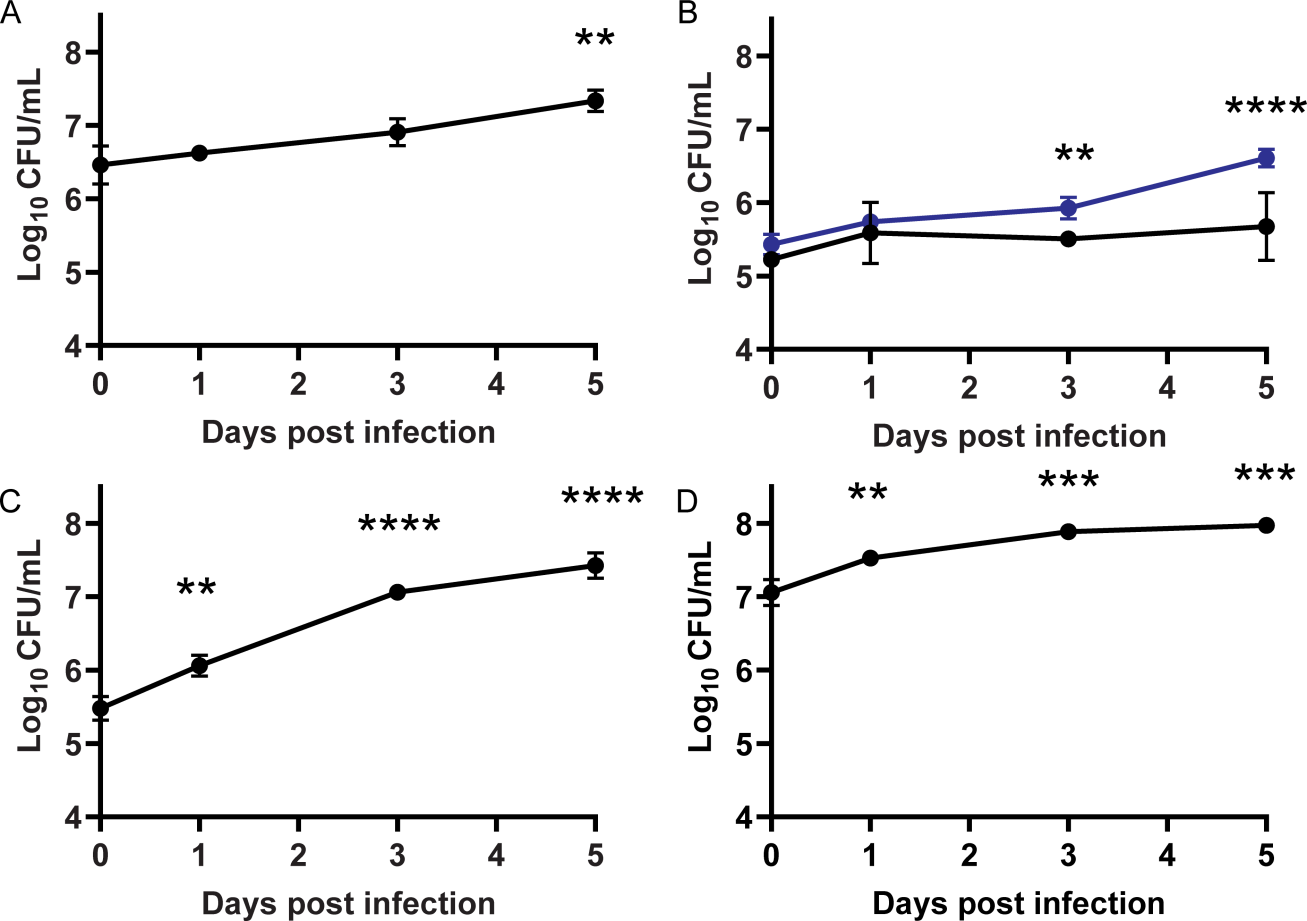
Clinical *M. abscessus* strains grow in mammalian cells. Mammalian cells were infected with *M. abscessus* strains at an MOI of 10. (**A**) THP-1 cells infected with *M. abscessus* GD82. (**B**) THP-1 cells infected with *M. abscessus* GD20 (blue) and *M. abscessus* ATCC 19977 Rough (black). (**C**) A549 cells infected with GD82. (**D**) Murine BMDM infected with GD82. Triplicate wells of infected mammalian cells were lysed for each time point between 0 and 5 days post infection and plated for CFU enumeration. Data shown are representative of two independent experiments. **P < 0.01, ***P < 0.001, ****P < 0.0001 by one-way ANOVA with Tukey’s post hoc test when compared to day 0.

While *M. abscessus* strains are shown to survive and grow in macrophages, fibroblasts, and lung epithelial cells (28, 56–59), GD82 and GD20 were not previously evaluated in any mammalian cells. To confirm that these strains survive intracellularly, we tested them in cultured macrophages and lung epithelial cells. Infections were performed with an MOI of 10 for 3 h before extensive washing to remove extracellular bacteria. After the final wash, fresh media with amikacin was added to the infected monolayer to inhibit growth of extracellular *M. abscessus*. At specific time points, cell monolayers were washed, lysed, and plated to enumerate intracellular bacterial CFU. GD82 and GD20 showed significant intracellular growth over a 5-day time course in the human THP-1 monocyte-derived macrophage cell line (Fig. 2A and B). In contrast to the growth seen with GD82 and GD20, ATCC 19977 Rough survived but did not significantly grow in THP-1 cells (Fig. 2B). GD82 was additionally evaluated in primary murine bone marrow derived macrophages (BMDM) and in human A549 lung epithelial cells. In both BMDM and A549 cells, GD82 CFU increased over time with the greatest degree of intracellular growth for this strain being observed in A549 cells (Fig. 2C and D).

### Mammalian cells internalize phage

**Fig 3:**
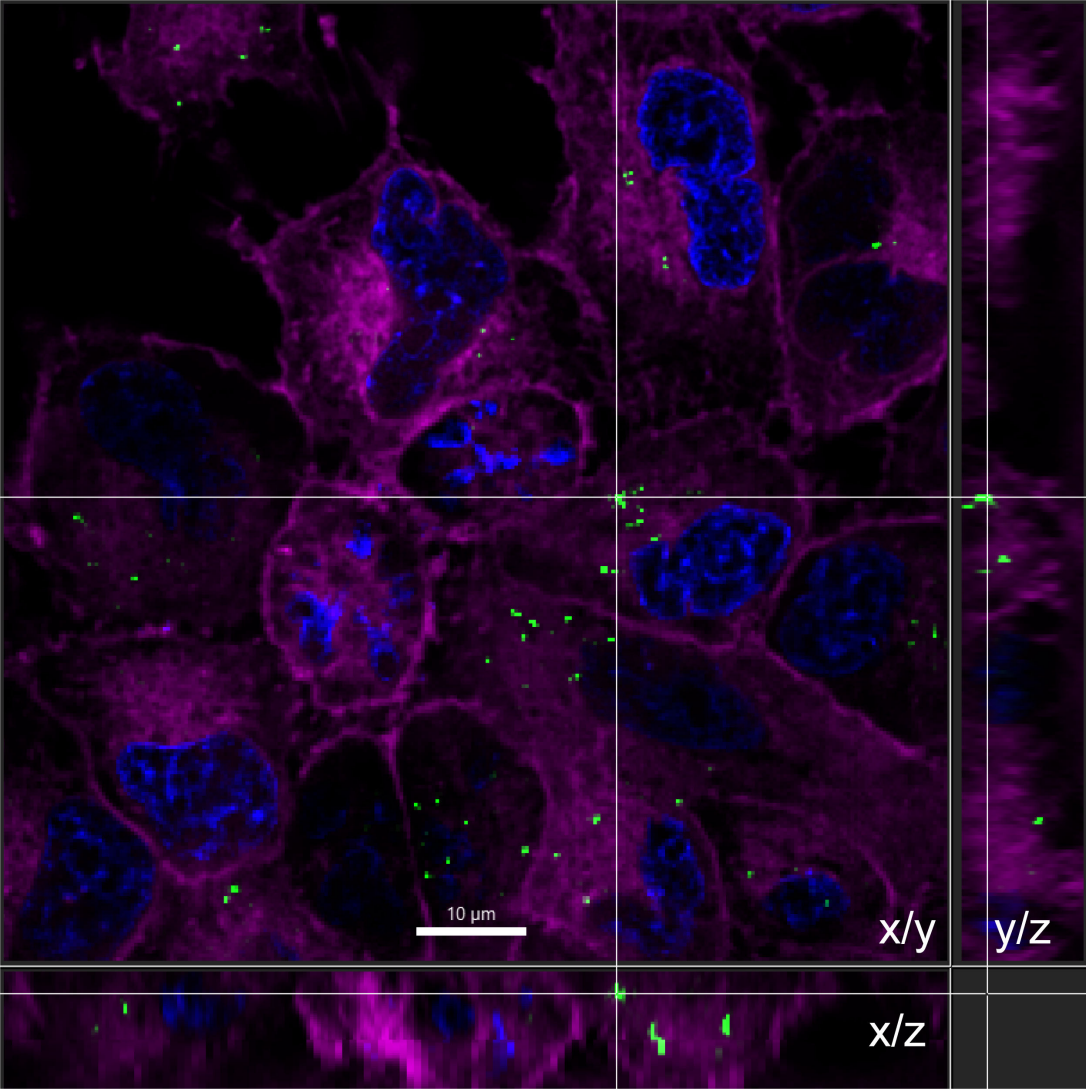
SYBR Gold phage are internalized by A549 cells. A 3D deconvoluted Z-series was used to generate an orthogonal view of BPsΔ (MOI 10^5^) inside A549 cells. Phage were stained with SYBR Gold (green). A549 cells were stained with CellMask plasma membrane (magenta) and DAPI (blue). Side views represent the Z dimension. Scale bar is 10 µm.

**Fig 4:**
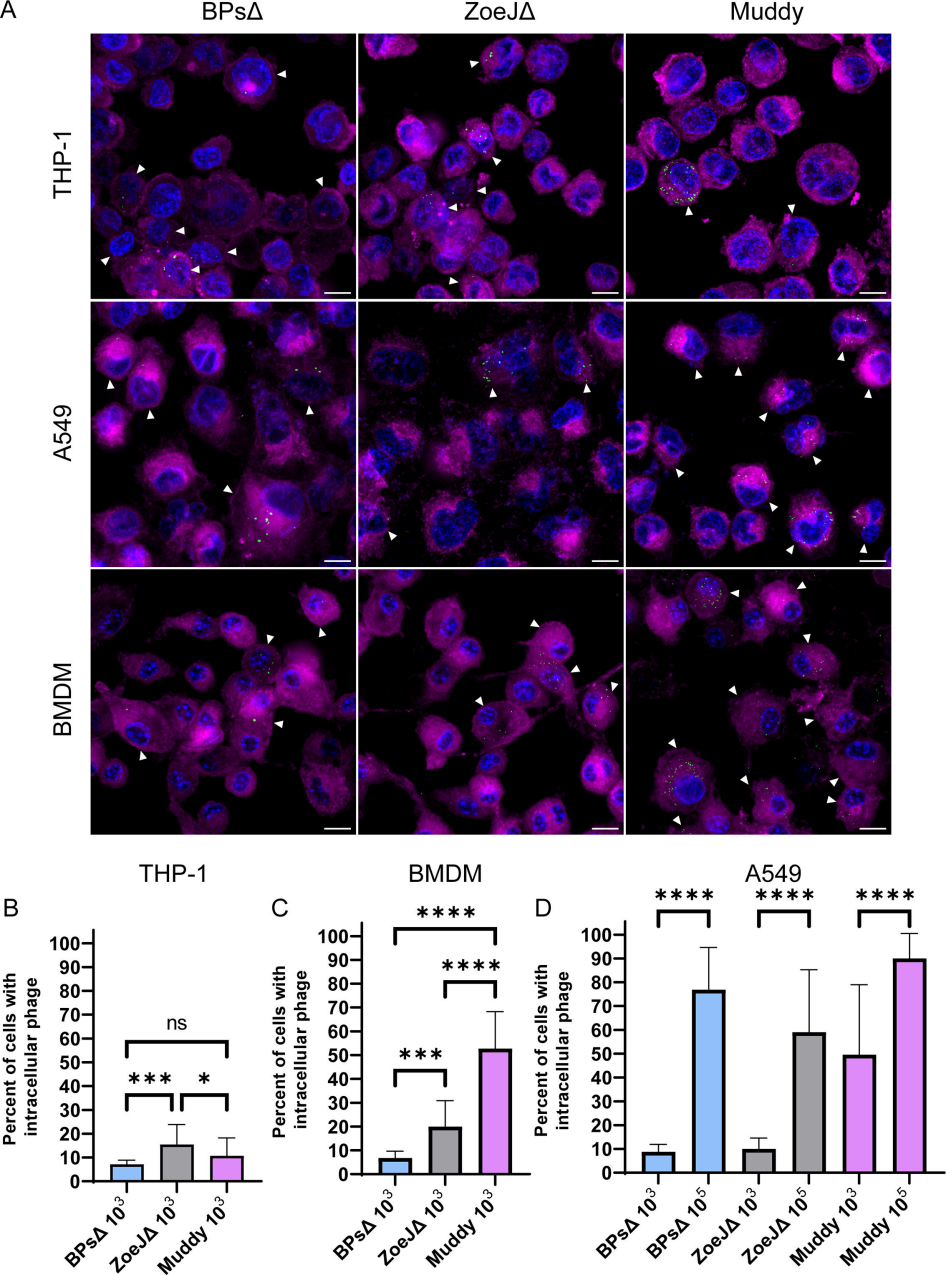
Mammalian cells internalize phage. Phages were stained with SYBR Gold and incubated with mammalian cells for 24 h. (**A**) Representative images of intracellular phage (green) at an MOI of 10^3^. Mammalian cells were stained with CellMask plasma membrane (magenta) and DAPI (blue). White arrows indicate mammalian cells with intracellular phage puncta. Scale bar is 10 µm. Percentage of THP-1 cells (**B**), BMDMs (**C**), and A549 cells (**D**) with intracellular phage. For B and C, phage were added to mammalian cells at an MOI of 10^3^. For D, phage were added at an MOI of 10^3^ or 10^5^. Error bars represent standard deviation from two independent experiments, each with a minimum of 14 fields of view counted. A minimum of 300 mammalian cells were counted per experiment. ns, not significant; *P < 0.05, ***P < 0.001, ****P < 0.0001 by Kruskal–Wallis one-way ANOVA with Dunn’s post hoc test.

The extent to which mammalian cells internalize phage will have a significant impact on the efficacy of phage therapy for intracellular bacteria like *M. abscessus*. Using THP-1, BMDM, and A549 cells, we measured uptake of SYBR Gold-stained BPsΔ, ZoeJΔ, and Muddy phage particles. Prior to each experiment, we measured the titer of stained phage using a plaque assay. Importantly, SYBR Gold staining did not reduce phage infectivity (Fig. S1). SYBR Gold-labeled phages were incubated with mammalian cells for 24 h at an MOI (phage:mammalian cells) of 10^3^ or 10^5^, after which time the cell monolayers were washed and stained with CellMask plasma membrane stain and DAPI to stain the nucleus. Using widefield fluorescence microscopy and 3D deconvolution, Z-stacks were taken, and 3D visualization revealed intracellular phage (i.e., phage puncta within the boundary of the plasma membrane stain) (Fig. 3). A maximum intensity Z projection was used to display all Z-stacks, and SYBR Gold-labeled phage particles were observed inside all three cell types (Fig. 4). At the lower MOI of 10^3^, BPsΔ was taken up at similar levels across the three cell types, with 7%–9% of cells in a field of view exhibiting intracellular BPsΔ phage puncta. For ZoeJΔ at the lower MOI, 10%–20% of the three cell types took up phage particles (Fig. 4). In contrast to these results, Muddy exhibited higher uptake and greater variation between cell types with 11% of THP-1 cells, 53% of BMDM, and 50% A549 cells harboring Muddy. With A549 cells, we also tested a higher MOI of 10^5^ and saw a significant increase in the percentage of cells with intracellular phage compared to the low MOI for all three phage (Fig. 4D). At the higher MOI, BPsΔ, ZoeJΔ, and Muddy were associated with 77%, 59%, and 90% of A549 cells, respectively. These experiments demonstrate the ability of these three phages to enter three mammalian cell types, while also revealing differences in uptake depending on cell type, phage, and MOI.

### Phage infection of intracellular *M. abscessus*

**Fig 5:**
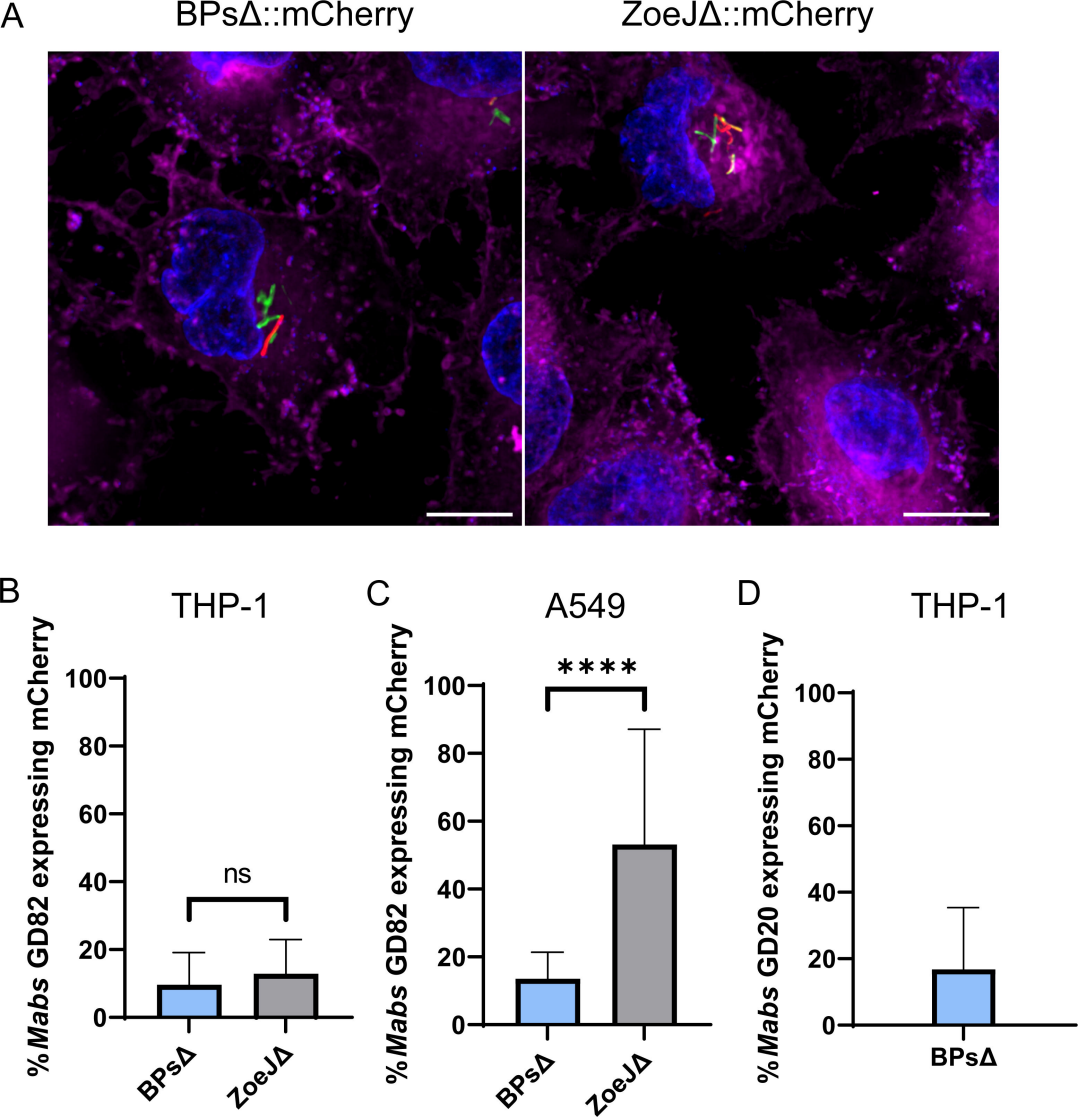
mCherry reporter phages infect intracellular mycobacteria. (**A**) Representative images of mCherry reporter phage infection of intracellular GD82 in A549 cells. A549 cells were infected with GFP-expressing GD82 at an MOI of 10, washed to remove extracellular bacteria, infected with BPs∆::mCherry or ZoeJΔ::mCherry phage at an MOI of 10^4^, and imaged after 24 h. Mammalian cells were stained with CellMask plasma membrane (magenta) and DAPI (blue). Scale bar is 10 µm. Red bacilli indicate phage-infected *M. abscessus*. Percent reporter phage infection of intracellular *M. abscessus* GD82 in THP-1 cells (**B**), GD82 in A549 cells (**C**), and GD20 in THP-1 cells (**D**). Error bars represent standard deviation from two independent experiments each with a minimum of 12 fields of view counted per experiment. A minimum of 350 *M*. *abscessus* cells (GFP) were counted per experiment. ns, not significant; ****P < 0.0001 by Mann–Whitney test.

To visualize phage-infected *M. abscessus* in the intracellular environment, we used BPsΔ and ZoeJΔ mCherry reporter phages, which carry the *mCherry* gene in their phage genome (49). These reporter phages identify phage-infected bacteria because *mCherry* is only expressed by bacteria following delivery of phage DNA into *M. abscessus* (49). Thus, the phage particles do not fluoresce, but phage-infected bacteria do. To detect intracellular phage infection, mammalian cells were first infected with GFP-expressing *M. abscessus* at an MOI of 10 for 3 h. Then, after extracellular bacteria were extensively washed away, BPsΔ mCherry or ZoeJΔ mCherry phage were added at an MOI of 10^4^ (phage:mammalian cells) in fresh media containing amikacin. After 24 h of incubation with phage, the cell monolayers were washed and imaged. By acquiring Z-stacks and using 3D visualization, we confirmed the presence of intracellular phage-infected bacteria (Fig. S2). In both THP-1 and A549 cells, mCherry-positive rod-shaped *M. abscessus* were observed, indicating intracellular phage-infected bacteria (Fig. 5; Fig. S3). As a control, when mCherry reporter phage were added to THP-1 cells alone, with no *M. abscessus*, no mCherry signal was observed (Fig. S4). To measure phage-infected *M. abscessus*, we counted green (GFP) and red (mCherry) intracellular *M. abscessus* (Fig. 5). In THP-1 cells, 10% and 13% of *M. abscessus* GD82 bacteria were mCherry positive (i.e., infected by phage), following treatment with BPsΔ mCherry or ZoeJΔ mCherry, respectively. In A549 cells, 14% and 53% of GD82 were mCherry positive following treatment with BPsΔ mCherry or ZoeJΔ mCherry, respectively. Phage-infected intracellular bacteria were also observed with *M. abscessus* GD20-infected THP-1 cells and BPsΔ mCherry (Fig. 5). These results demonstrate the ability of two different phages to infect two different intracellular *M. abscessus* strains in two different mammalian cell types.

### Transmission electron microscopy (TEM) of phage and *M. abscessus* in mammalian cells

**Fig 6:**
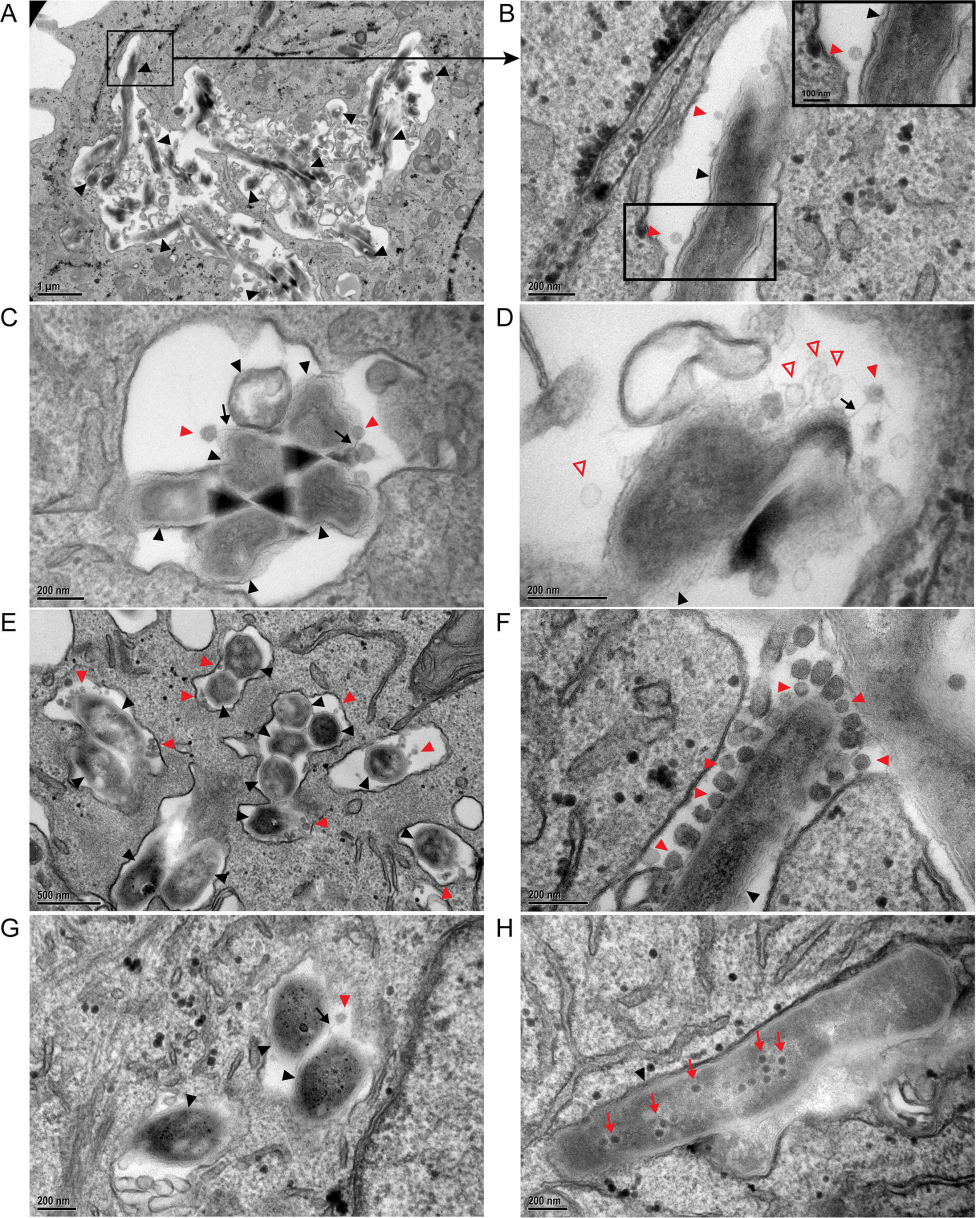
Transmission electron microscopy of phage infection of intracellular *M. abscessus*. GD20-infected THP-1 cells were incubated with BPsΔ for 24 h (**A–D**). THP-1 phagosome with BPsΔ phage adsorbed to intracellular GD20 (**A and B**). BPsΔ phage tails adsorb to intracellular GD20 (**C**). BPsΔ phage, with empty and intact capsids, adsorbed to intracellular GD20 (**D**). GD82-infected THP-1 cells were incubated with BPsΔ for 24 h. Colocalized phage and bacteria are observed in multiple phagosomes in the same cell (**E**). GD82-infected THP-1 cells incubated with BPsΔ for 48 h. Multiple phages adsorbed to the bacterial pole are observed (**F**). GD82-infected A549 cells incubated with BPsΔ for 24 h and a phage tail adsorbed to intracellular bacteria (**G**). GD82-infected A549 cells were incubated with BPsΔ for 48 h, and intracellular phage progeny was observed (**H**). Black arrowheads indicate intracellular *M. abscessus*, red arrowheads indicate adsorbed phage, black arrows indicate phage tails, red outlined arrowheads indicate empty phage capsid, and red arrows indicate phage progeny. Only a subset of phage particles and bacteria are indicated. Black boxes indicate areas of higher magnification (**A and B**). For additional TEM images, see Fig. S5.

We used TEM to observe phage and *M. abscessus* in mammalian cells at a higher resolution. Using the same infection protocol as with the reporter phage experiments, THP-1 or A549 cells were first infected with *M. abscessus* GD20 or GD82 followed by treatment with BPsΔ phage at an MOI of 10^4^ for 24 or 48 h. In all these experiments, a subset of *M. abscessus*-containing phagosomes harbored structures matching the capsid shape and size (55 nm) of BPs phage (60). Many of these phage particles were closely associated with *M. abscessus* and appeared to be interacting with the bacteria (Fig. 6). In some cases, we observed phage tails that appeared to be adsorbed to intracellular bacteria (Fig. 6C, D, and G; Fig. S5D). In other cases, we observed phage particles that were closely associated with intracellular bacteria but possibly in an orientation that did not allow visualization of tails (Fig. 6B and F; Fig. S5F). Potential examples of empty phage capsids, with a less electron-dense capsid, were also observed, plausibly representing phage particles that had completed the DNA delivery step of phage infection (Fig. 6D). These putative empty heads appeared slightly larger than intact capsids; however, this is most likely a reflection of different staining of empty and intact heads. Additionally, we observed some examples of phage particles congregating at the cell pole and septal regions of the bacteria (Fig. 6F), consistent with other reports of *Mycobacterium* phage infection (61). Finally, after a longer period of phage treatment (48 h), we observed examples of intracellular *M. abscessus* with electron-dense particles resembling phage capsids inside the bacterial cell (Fig. 6H). These particles are reminiscent of previously described phage assembly domains in bacterial cells, which may reflect phage replication in intracellular *M. abscessus* (61, 62). These findings agree with and reinforce the results of the mCherry reporter phage experiments (Fig. 5) in demonstrating co-localization of phage and *M. abscessus* in phagosomes of THP-1 and A549 cells in a manner consistent with ongoing phage infection.

### Phage kill intracellular *M. abscessus* in a cell type-dependent manner

**Fig 7:**
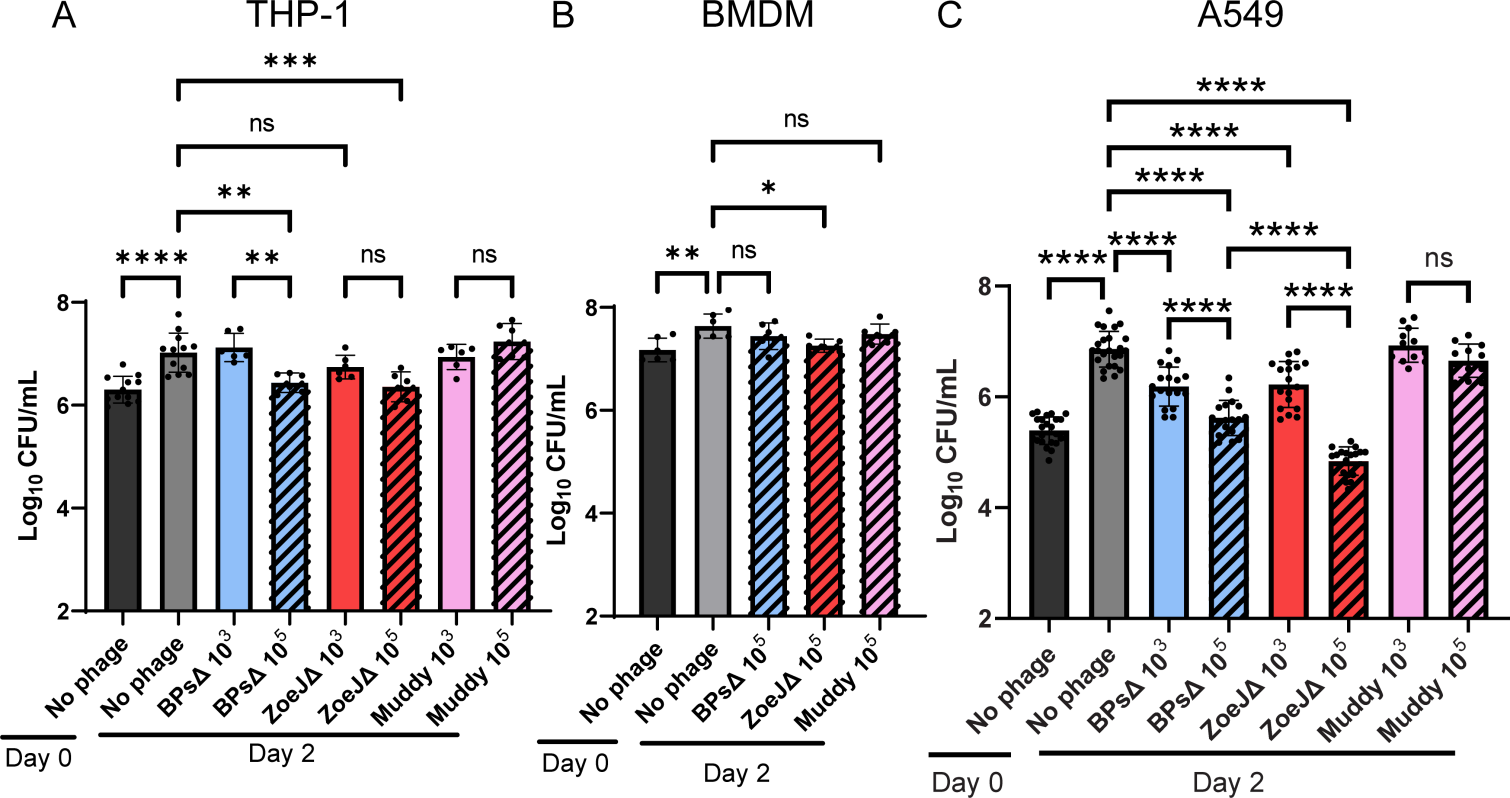
Phages kill intracellular *M. abscessus* GD82 in mammalian cells. (**A**) THP-1 cells, (**B**) BMDM, or (**C**) A549 cells were infected with *M. abscessus* GD82 at an MOI of 10, washed to remove extracellular GD82, and then treated with BPsΔ, ZoeJΔ, or Muddy. After 48 h, mammalian cells were lysed in the presence of PIB and plated for bacterial CFU enumeration. Error bars represent standard deviation from a minimum of two independent experiments determined by one-way ANOVA with Tukey’s post hoc test; ns, not significant; *P < 0.05, **P < 0.01, ***P < 0.001, ****P < 0.0001.

Lastly, we addressed whether phage treatment of intracellular *M*. *abscessus* impacts intracellular CFU. Mammalian cells were first infected with *M. abscessus* GD82 and subsequently treated, as before, for 2 days with phage at an MOI of 10^3^ or 10^5^. After phage treatment, monolayers were washed, lysed in the presence of a PIB, and plated to enumerate intracellular CFU. PIB treatment was included to prevent any free phage in the lysate from infecting *M. abscessus* during outgrowth of bacteria on agar. As shown in the supplemental data, PIB treatment with 0.1% Triton X-100 inactivated phage but had no impact on *M. abscessus* CFU (Fig. S6). In the absence of phage, there was a significant increase in *M. abscessus* CFU over 2 days in all three cell types (Fig. 7). As seen previously, GD82 growth was greatest in A549 cells (Fig. 7C). In THP-1 cells at the lower MOI, none of the three phages had an effect on GD82 intracellular CFU. However, with the higher MOI, treatment with BPsΔ or ZoeJΔ phage led to a significant decrease (0.6 log) in GD82 CFU compared to untreated GD82-infected THP-1 cells (Fig. 7A). In contrast, Muddy did not reduce CFU levels even at the higher MOI in THP-1 cells. In BMDMs, only the high MOI was tested. In BMDMs, the high MOI of ZoeJΔ led to a significant, albeit modest, reduction in the level of intracellular CFU (0.3 log) (Fig. 7B), while BPsΔ and Muddy had no effect. Finally, in A549 cells, both low and high MOIs of BPsΔ or ZoeJΔ significantly reduced intracellular CFU compared to untreated GD82-infected cells. The effect was dose dependent (Fig. 7C), and of the two phages, ZoeJΔ had the more pronounced effect. The average decrease in CFU at the lower MOI was 0.7 log for both BPsΔ and ZoeJΔ. At the higher MOI, there was a 1.3-log reduction of CFU for BPsΔ and a 2.1-log reduction of CFU for ZoeJΔ. In A549 cells, Muddy again did not have an effect on intracellular *M. abscessus* (Fig. 7C). The lack of an effect for Muddy reveals differences between phages in their ability to function in an intracellular environment. These data demonstrate that some, but not all, phages from clinical cases are able to kill *M. abscessus* in mammalian cells. Furthermore, the mammalian cell type harboring the bacteria has an impact on the effect of the phage, with the most pronounced phage effect on *M. abscessus* CFU observed in lung epithelial A549 cells.

## DISCUSSION

The need for more effective therapies for *M. abscessus* infection has spurred interest in the use of phages as treatments for this drug-resistant pathogen. However, fundamental questions remain about the efficacy and feasibility of phage therapy for *M. abscessus* (30). As *M. abscessus* is a facultative intracellular pathogen, a critical unknown is whether phages can infect and kill intracellular *M. abscessus* residing in mammalian host cells (63). Moreover, it is often assumed that phage will not be internalized by or survive in mammalian cells (36–40). Here, using *M. abscessus* strains and phages from clinical cases, we demonstrated that phages are taken up by mammalian cells and are able to infect and kill intracellular *M. abscessus*. We additionally uncovered differences between mammalian cell types and individual phages in the efficiency of phage uptake, infection, and killing of intracellular *M. abscessus*.

All three SYBR Gold-labeled phages were internalized by mammalian cells. With BPsΔ and ZoeJΔ, the percentage of mammalian cells with intracellular phage particles was similar across three cell types. However, with Muddy, a higher percentage of A549 and BMDM cells harbored phage compared to THP-1 cells. In a study of *Escherichia coli* T4 phage uptake by mammalian cells, a higher percentage of A549 cells compared to THP-1 cells was similarly found to uptake phage (42). Furthermore, in that same study, the authors concluded that phage size correlates with the efficiency of mammalian cell uptake (42). In our study, Muddy exhibited the greatest level of intracellular localization. However, the phages in our study are similarly sized siphoviruses (50, 60). Therefore, instead of size, we speculate that Muddy has other distinctive features, such as the molecular composition of its surface or properties critical to phage persistence inside mammalian cells, which account for its higher association with certain cell types. With A549 cells, we tested phage at two MOIs (10^3^ and 10^5^) and saw a dose-dependent increase in the percentage of cells with intracellular phage. Thus, with the lower MOI, cellular uptake was not yet saturated in our cell culture system. To give these MOIs some context, *M. abscessus*-infected phage therapy patients are routinely given doses of 10^9^–10^10^ PFU of phage twice daily (30), and although it is hard to estimate the total bacterial burden in patients, sputum samples from the GD82-infected patient contained 10^3^ CFU/mL (32).

Using BPsΔ and ZoeJΔ mCherry reporter phages (49), we went on to visualize phage-infected *M. abscessus* in THP-1 and A549 cells. Muddy has proven intractable to current engineering strategies, and an mCherry reporter phage is not available. In these experiments, it is important to recognize that the probability of any mammalian cell carrying both phage and *M. abscessus* is significantly less than 100%, since not all mammalian cells will contain intracellular phage (Fig. 4). Additionally, the single time point chosen for analysis may have impacted the number of mCherry-positive bacteria detected. Regardless of these caveats, we detected a seemingly robust level (~10% or 50%) of intracellular *M. abscessus* bacteria being phage infected.

Consistent with the results from the reporter phage experiments, TEM analysis of *M. abscessus-*infected and phage-treated THP-1 and A549 cells revealed phagosomes containing phage and bacteria. This co-localization was observed with two *M. abscessus* strains. TEM revealed phage and bacterial interactions suggestive of different steps of the phage lifecycle: phage adsorption, injection of phage DNA, and production of phage progeny. We saw phage particles that appear to be adsorbed to intracellular bacteria. Some of these phage particles appear to have empty capsids, suggesting that phage infection had occurred. We also observed *M. abscessus* with dense internal structures reminiscent of newly synthesized phage particles, indicating ongoing and active phage replication. When phage and bacteria co-localized in a mammalian cell, they were found in large spacious phagosomes. We do not know if this reflects the ease of detecting phages in a more spacious phagosome or if there is a preference for phage particles to traffic to larger phagosomes. When uninfected THP-1 cells (i.e., no bacteria) were phage treated, it was difficult to detect phages inside cells, but in the few cases where they were observed, they were also in large spacious vacuoles (Fig. S5A). It is important to note that in addition to *M. abscessus* in spacious phagosomes, we also observed *M. abscessus* in tightly apposed phagosomes (Fig. S5G). Both GD82 and GD20 have a rough colony morphology, which is previously associated with tightly apposed phagosomes in BMDM (28).

The reporter phage and TEM experiments utilized mammalian cells that were first infected with *M. abscessus* and later extensively washed to remove extracellular bacteria and treated with phage alongside amikacin. Amikacin, which does not penetrate mammalian cells at a significant rate and has limited intracellular activity (64–67), was included to eliminate extracellular bacteria. Thus, we think it likely that the phage-infected intracellular bacteria observed are a reflection of phage particles that entered mammalian cells and then infected *M. abscessus* in the intracellular environment. Due to their small size, we speculate that phages are taken up by an endocytic pathway, rather than phagocytosis (68, 69), and that subsequent vacuole fusion events may be responsible for phage delivery to *M. abscessus*-containing phagosomes. However, we cannot rule out the possibility that phage infection of a small population of extracellular bacteria occurred and that these phage-infected bacteria were subsequently internalized by mammalian cells. While we consider this latter scenario less likely, mammalian cell uptake of phage-infected extracellular *M. abscessus* could also be a route for phage delivery during *in vivo* infection (45, 46).

Most significantly, we observed an effect of phage on intracellular *M. abscessus* CFU. Among the three mammalian cell types assessed, the greatest killing occurred in A549 epithelial cells, while the two macrophage cell types exhibited modest to no phage killing. Currently, we do not have an explanation for the different effects in lung epithelial cells versus macrophages. One possibility is that the metabolic state of *M. abscessus* differs in these cell types and this impacts phage lytic activity. In support of this possibility, there are reports of replicating bacteria being more conducive for phage replication and bacterial lysis, which is interesting considering that GD82 grew more efficiently in A549 cells than in macrophages (Fig. 2) (70–72). In addition to the difference in CFU reduction that depended on mammalian cell type, we observed differences in the ability of individual phage to kill intracellular *M. abscessus*. While both BPsΔ and ZoeJΔ significantly reduced GD82 CFU in A549 cells, ZoeJΔ exhibited the greater effect. Consistent with this observation, in A549 cells, ZoeJΔ mCherry phage also infected intracellular *M. abscessus* to a greater degree than BPsΔ mCherry (Fig. 5). Muddy, however, did not exhibit any significant effect on CFU in any cell type, even at the higher MOI. This is interesting because SYBR Gold-labeled Muddy exhibited the highest intracellular localization of the three phages (Fig. 4). One possibility for the lack of intracellular killing by Muddy is that the bacterial receptor that Muddy recognizes is not expressed in the intracellular environment. Further study is required to address this possibility. It is noteworthy that we included a phage inactivation step (i.e., PIB treatment) prior to plating lysates to enumerate CFU (43). The inclusion of this step gives us confidence that the CFU differences observed reflect phage killing that occurred during the phage treatment of intracellular *M. abscessus* and not during *M. abscessus* recovery from infected cells.

The CFU data demonstrate that phages can not only enter mammalian cells but also infect and kill intracellular *M. abscessus*. Although the potential for phages to act on intracellular bacteria is not extensively studied, there are reports of phage killing of intracellular bacteria, as reviewed by Goswami et al. (73). Some of these examples use free phage (43, 44, 74), as we did in this study, while others utilize phage delivered by phage-infected bacteria in a “Trojan horse” type of approach (45, 46). One noteworthy “Trojan horse” study involved mycobacteriophage TM4 and *Mycobacterium avium*- and *Mycobacterium tuberculosis*-infected macrophages (45). In that study, Broxmeyer et. al found that addition of free phage particles to *M. avium*-infected RAW 264.7 cells had no effect on intracellular CFU. However, treatment with previously phage-infected mycobacteria led to a reduction in intracellular CFU (45). Given that our results differ in demonstrating free phage to exert a killing effect on intracellular mycobacteria, it is important to note that we used different phage, higher MOIs, and different mycobacteria species than Broxmeyer et al. Moreover, we found the most pronounced effect on intracellular CFU in A549 epithelial cells as opposed to macrophages, which were the cell type used by Broxmeyer et al.

In summary, here, we establish the potential for clinically relevant phages to be taken up by mammalian cells and for intracellular *M. abscessus* to be infected and killed by phage. A key to improving phage therapy could come from understanding the differences that we uncovered between phages and cell types that impact the ability of phage to kill intracellular *M. abscessus*. So far, phage therapy candidates have only been tested *in vitro* for the ability to lyse bacteria. Future candidates could also be screened for their ability to kill intracellular *M. abscessus*. Additionally, methods to increase phage uptake, such as liposomal encapsulation and polymer modification, may have the potential to improve phage delivery into mammalian cells and further improve efficacy (75–78).

## Data Availability

Data from this study are available through Dryad at https://doi.org/10.5061/dryad.t1g1jwt8h.

## SUPPLEMENTAL MATERIAL

Supplemental Figures mbio.02924-23-s0001.docx Fig. S1-S6.

Supplemental Methods mbio.02924-23-s0002.docx Additional experimental details.

ASM does not own the copyrights to Supplemental Material that may be linked to, or accessed through, an article. The authors have granted ASM a non-exclusive, world-wide license to publish the Supplemental Material files. Please contact the corresponding author directly for reuse.
