# Supplementary material for: Bacteriophage infection and killing of intracellular Mycobacterium abscessus
Source: mBio. 2023 Dec 7;15(1):e02924-23. doi: 10.1128/mbio.02924-23 (PMC10790704; doi:10.1128/mbio.02924-23)
Supplement: Supplemental Figures — Fig. S1-S6. [file mbio.02924-23-s0001.docx]

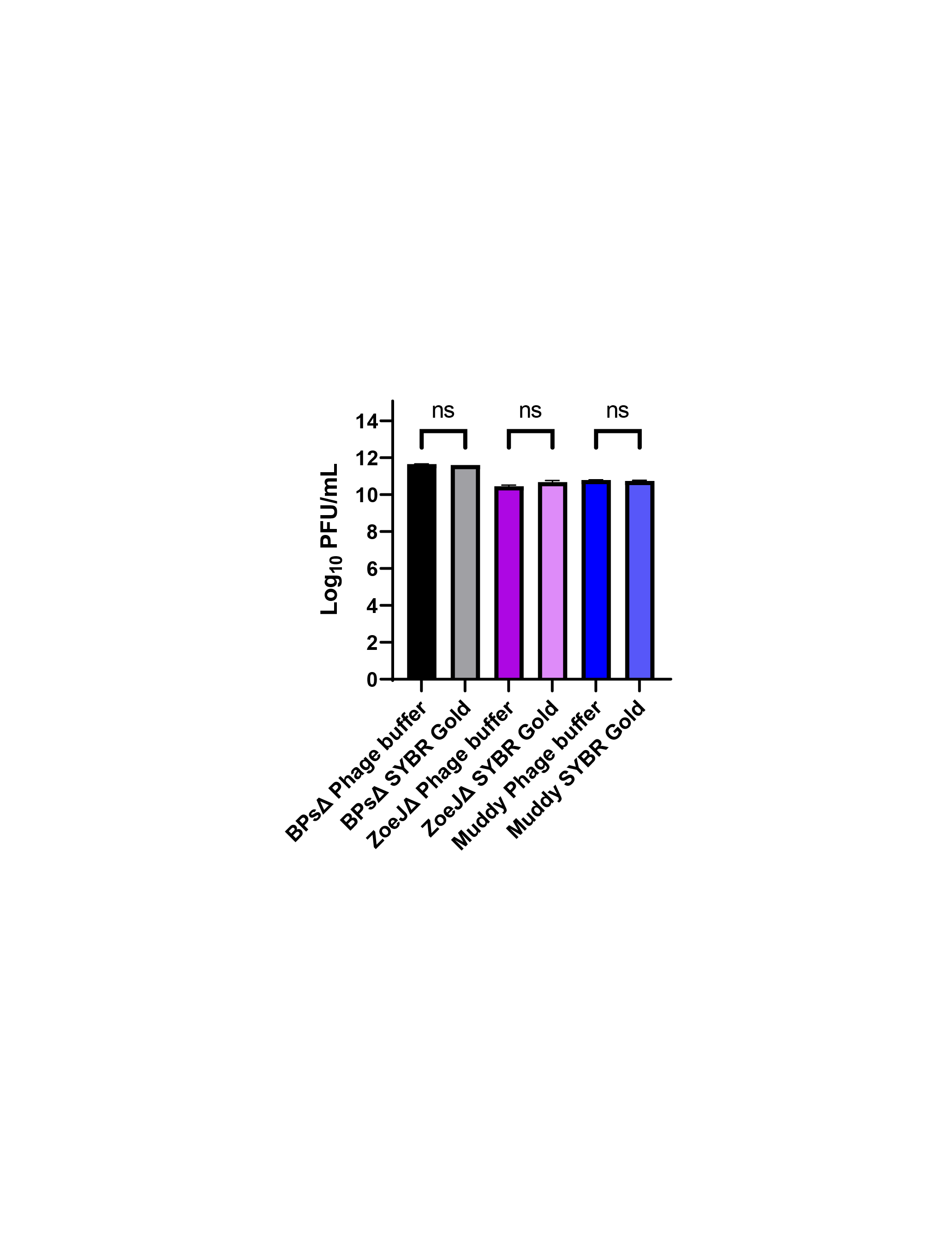


**Fig S1**. SYBR Gold staining does not inhibit phage activity. Phages were incubated in phage buffer or SYBR Gold for 1 hr at 4^o^ C and the phage titer was enumerated via plaque assay.


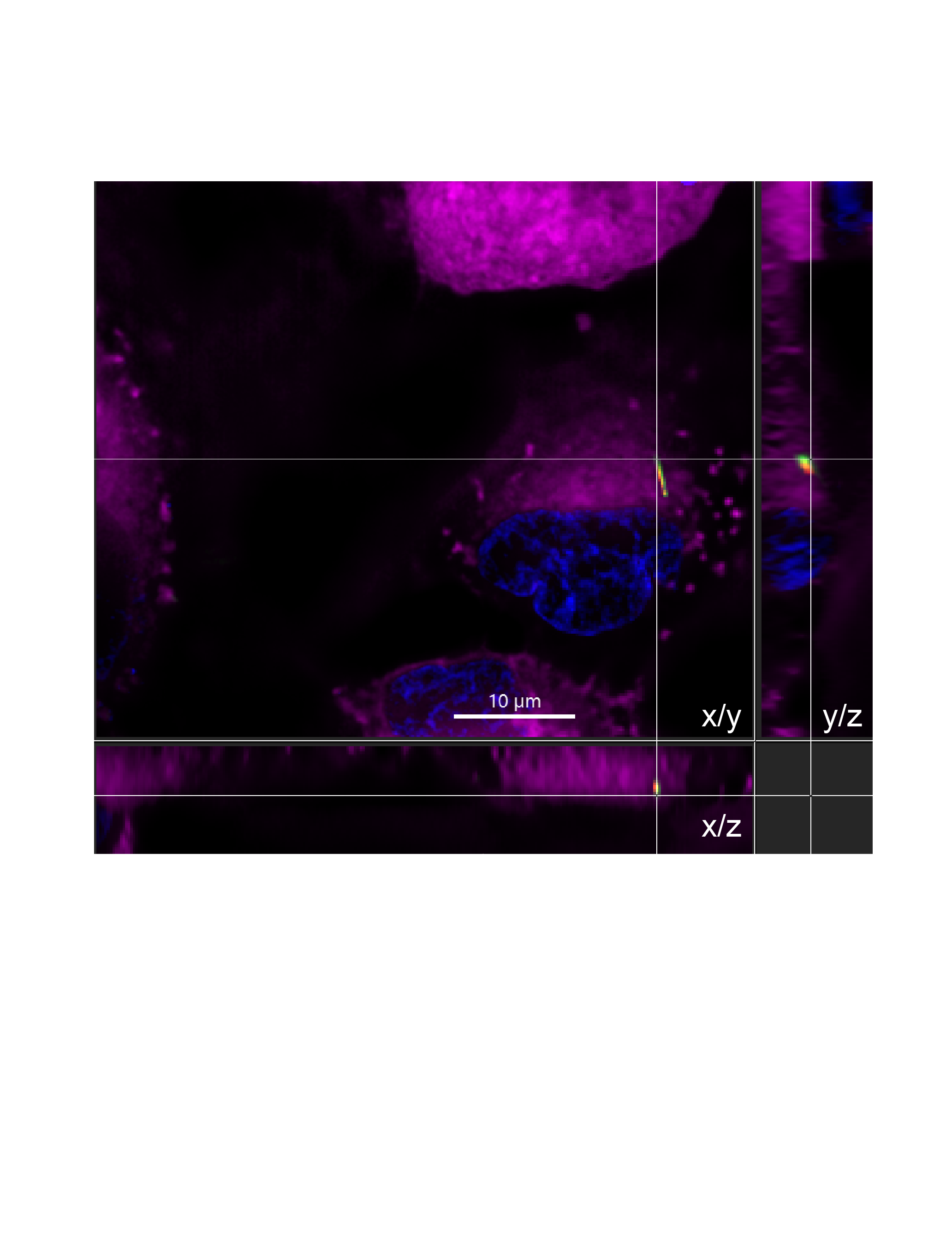


**Fig S2**. mCherry positive *M. abscessus* are intracellular. A 3D deconvoluted Z-series was used to generate an orthogonal view of ZoeJΔ::mCherry infecting intracellular GD82 in A549 cells. Side views represent the Z dimension. A549 cells were infected with GFP expressing GD82 at an MOI of 10, washed to remove extracellular bacteria, and infected with ZoeJΔ::mCherry phage at an MOI of 10^4^ and imaged after 24hrs. Red bacilli indicate phage-infected *M. abscessus*. A549 cells were stained with CellMask plasma membrane (magenta), and DAPI (blue).


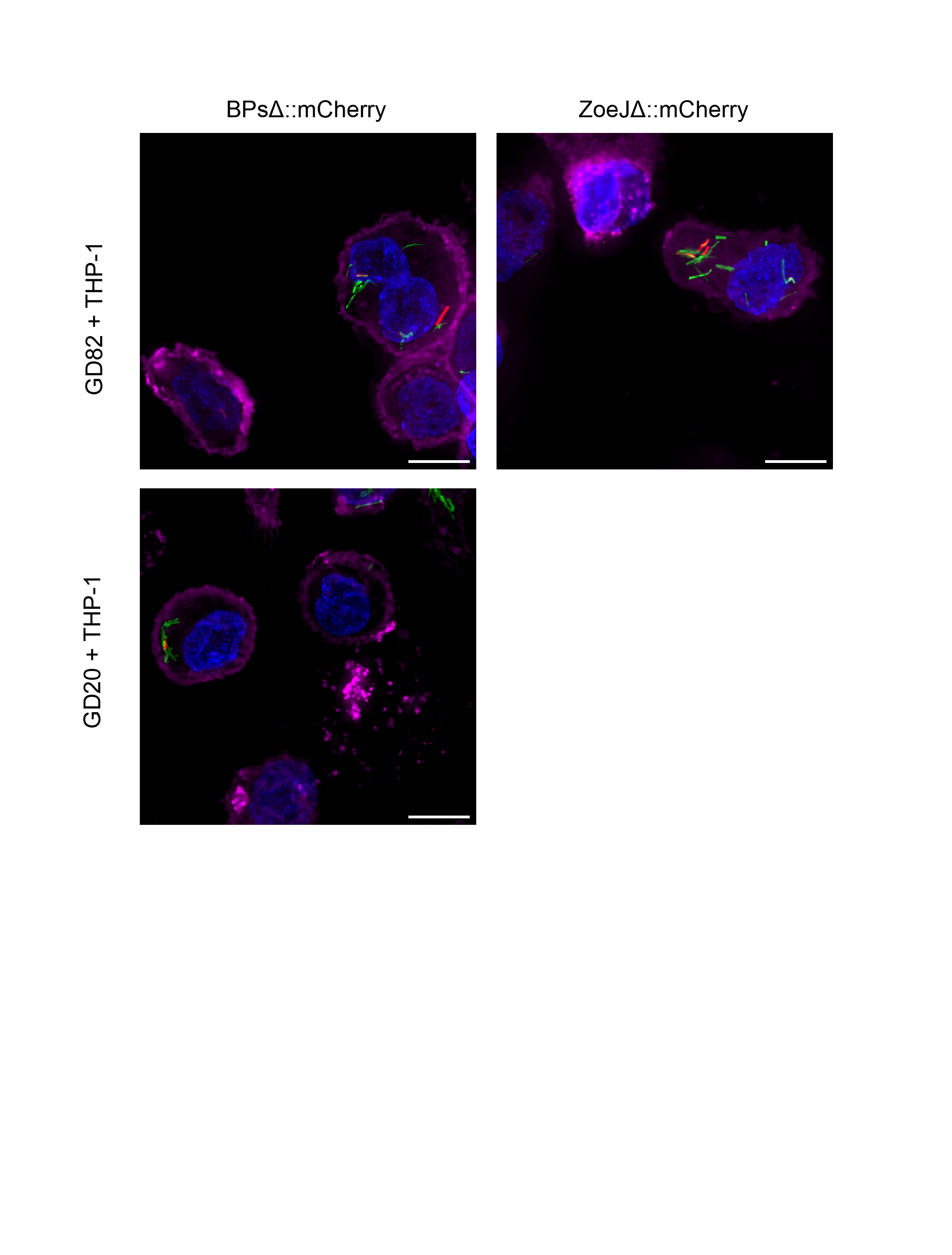


**Fig S3**. Reporter phage infects intracellular mycobacteria. Representative images of mCherry reporter phage infection of intracellular GD82 and GD20 in THP-1 cells. THP-1 cells were infected with GFP-expressing GD82 or GD20 at an MOI of 10, washed to remove extracellular bacteria, treated with BPs∆::mCherry or ZoeJΔ::mCherry phage at an MOI of 10^4^ and imaged after 24hrs. Red bacilli indicate phage-infected *M. abscessus*. Mammalian cells were stained with CellMask plasma membrane (magenta), and DAPI (blue). Scale bar is 10 μm.


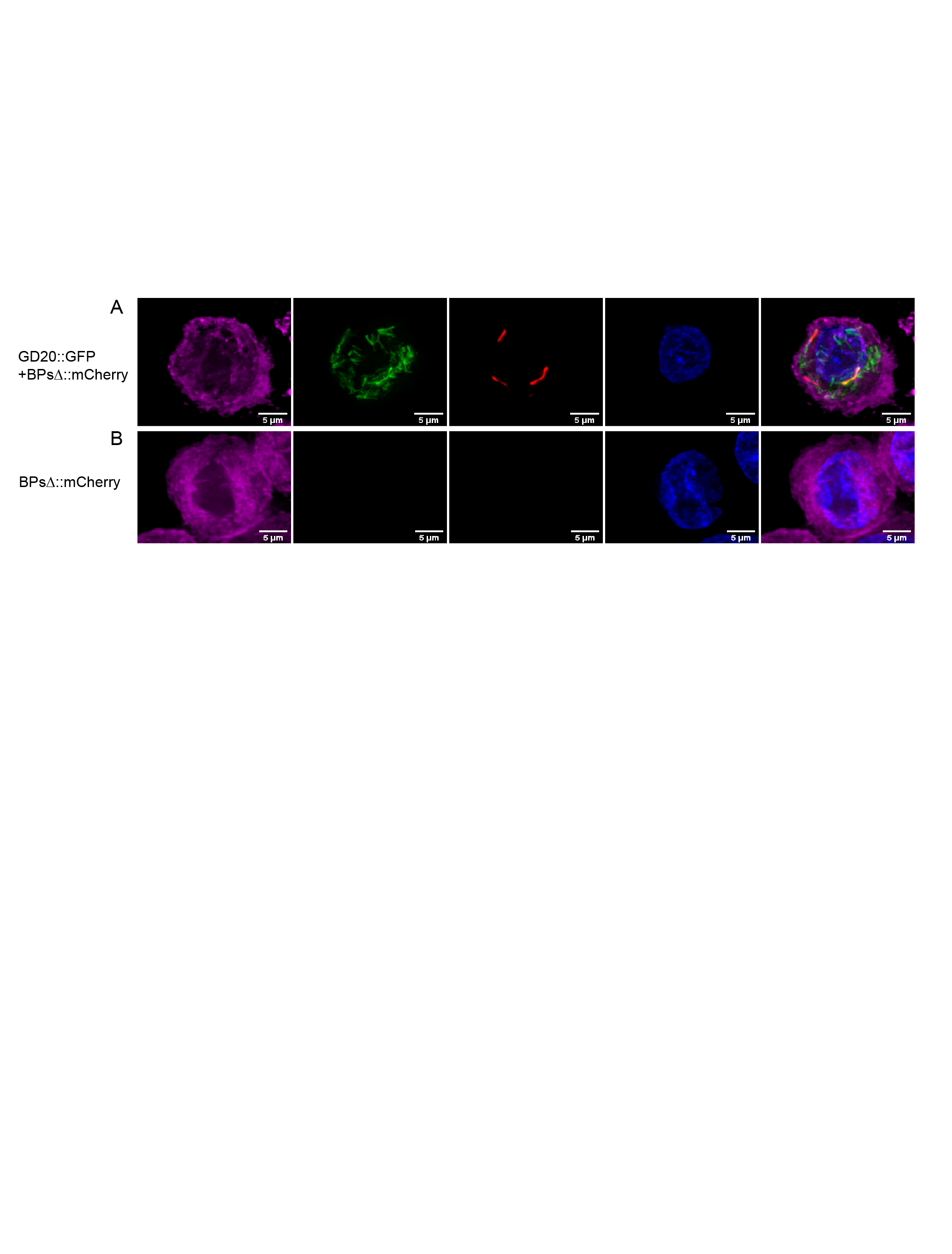


**Fig S4**. mCherry reporter signal depends upon *M. abscessus* infection. A) mCherry reporter phage infection of intracellular GD20 in THP-1 cells. THP-1 cells were infected with GFP expressing GD20 an MOI of 10, washed to remove extracellular bacteria, incubated with BPs∆::mCherry at an MOI of 10^4^, and imaged after 24hrs. Red bacilli indicate phage-infected *M.* abscessus. A representative image was chosen from a field of view of 41 cells. B) BPs∆::mCherry was added to THP-1 cells at an MOI of 10^4^ and imaged 24hrs later. THP-1 cells were stained with CellMask plasma membrane (magenta), and DAPI (blue). A representative image was chosen from a field of view of 32 cells.


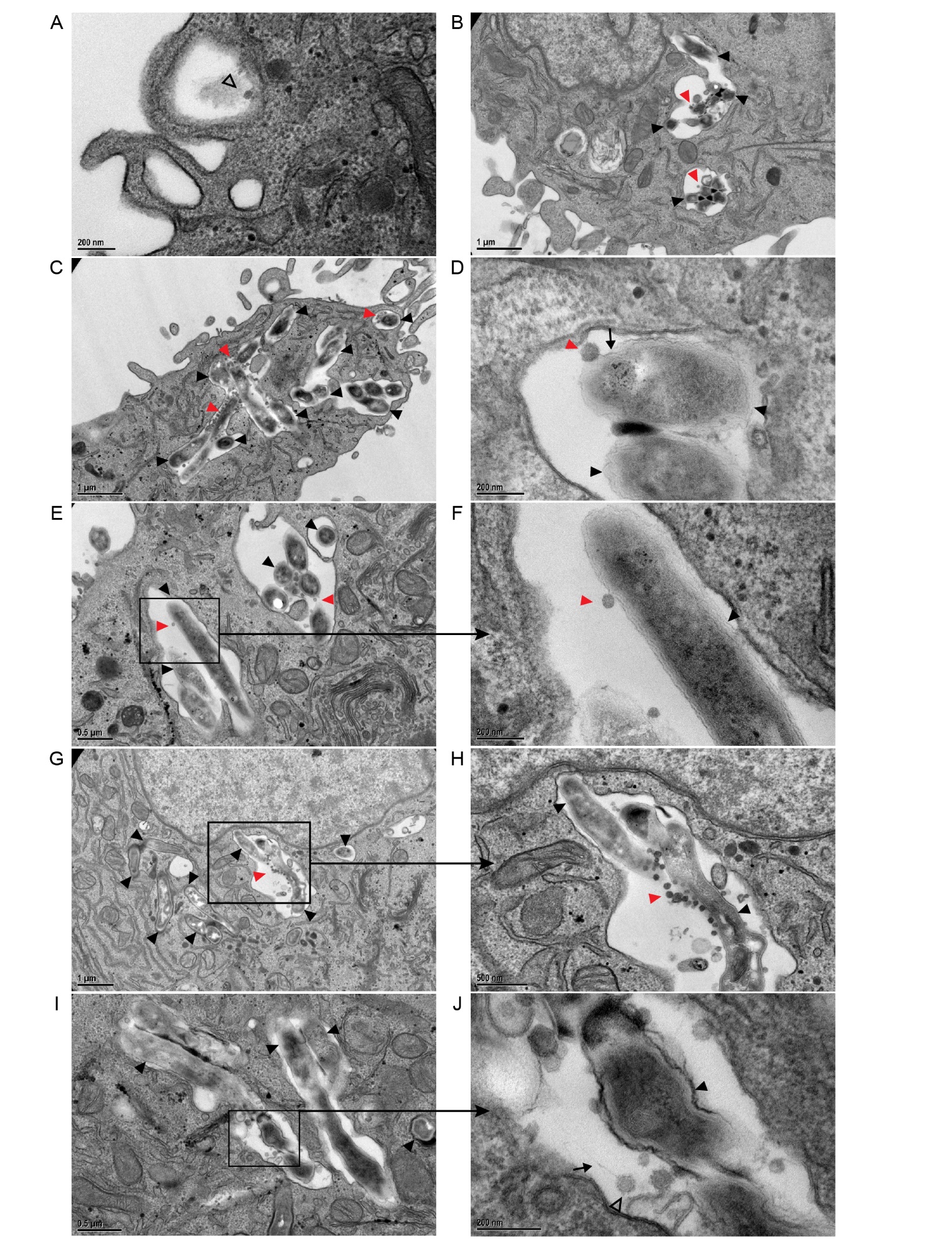


**Fig S5**. Additional transmission electron microscopy of phage infection of intracellular *M. abscessus*. Uninfected THP-1 cells were incubated with BPsΔ alone for 24hrs (A). GD20 infected THP-1 cells were incubated with BPsΔ for 24hrs (B, I, J). GD82 infected THP-1 cells were incubated with BPΔ for 48hrs (C, G, H) or 24hrs (D, E, F). Black arrowheads indicate intracellular *M. abscessus*, red arrowheads indicate adsorbed phage, black arrows indicate phage tails, black outlined arrowheads indicate unadsorbed intracellular phage. Only a subset of phage particles and bacteria are indicated. Black boxes indicate areas of higher magnification (E-J). B is a zoomed out image of Fig. 6C and C is a zoomed out image of Fig 6F.


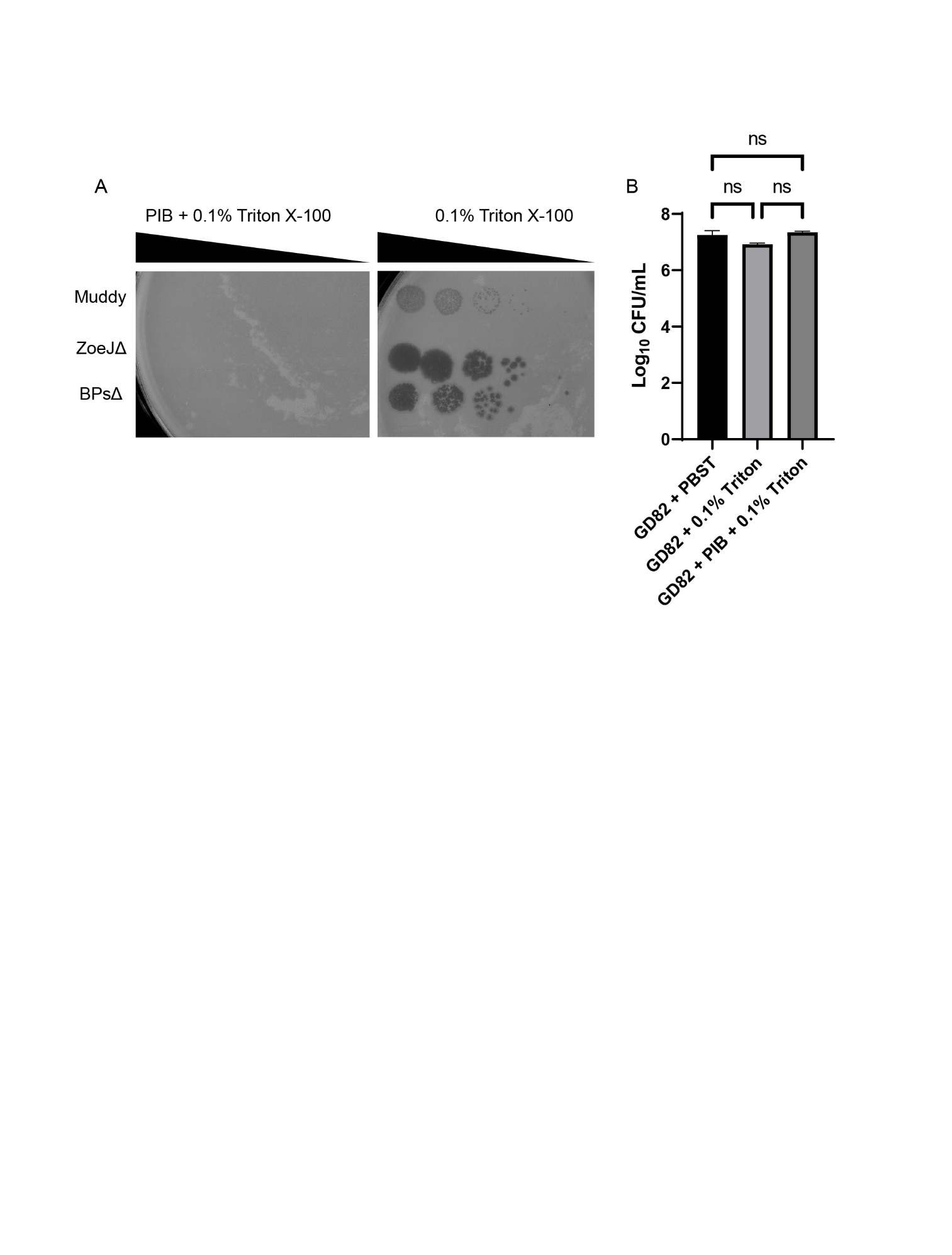


**Fig S6**. PIB inactivates phage but not *M. abscessus*. A) Phage and *M. abscessus* GD82 were incubated with 0.1% Triton X-100 +/- PIB for 10 min. Phage were serially diluted and spotted on *M. smegmatis* top agar plates. B) GD82 was incubated with 0.1% Triton X-100 +/- PIB or PBS with 0.05% Tween80 (PBST) for 10 min and CFUs were enumerated.
