# Supplementary material for: Bacteriophage infection and killing of intracellular Mycobacterium abscessus
Source: mBio. 2023 Dec 7;15(1):e02924-23. doi: 10.1128/mbio.02924-23 (PMC10790704; doi:10.1128/mbio.02924-23)
Supplement: Supplemental Methods — Additional experimental details. [file mbio.02924-23-s0002.docx]

**Supplemental Methods**

**Phage high titer lysate preparation**

Phage high titer lysates were prepared as previously described (1). Phages were propagated on lawns of *M. smegmatis*. Phage lysate was recovered by flooding plates with phage buffer (10mM Tris pH 7.5, 10mM MgSO_4_, 68mM NaCl, 1mM CaCl_2_) and sequentially filtering through 0.45 µm and 0.22 µm filters. The phage lysate was then precipitated overnight at 4^o^ C with 4% polyethylene glycol (PEG) 8000 and 0.5 M NaCl, final concentration. The precipitated phage was recovered by centrifugation for 30 min, 3,700 x g at 4^o^ C, and then resuspended in phage buffer overnight at 4^o^ C. Following a second centrifugation to separate phage from bacterial debris, the supernatant was transferred to an ultrafiltration centrifugation tube with a MWCO at 100kDa (Pierce Protein Concentrator, Thermo Fisher Scientific) centrifuged at 3,700 x g at 4^o^ C, and washed with 40 mL of phage buffer, as described previously (2). The purified lysate was then filtered through a 0.22 µM filter and stored at 4^o^ C.

**Bacterial strain construction**

*M. abscessus* strains were transformed via electroporation as previously described with modifications (3). *M. abscessus* was grown in 7H9 to an OD of ~1.5 with the addition of 0.2 M glycine 16hrs before harvesting cells. Cells were made electrocompetent by washing with 10% glycerol. After electroporation, cells were recovered in 7H9 for 16hrs and then plated on kanamycin 7H10 plates.

**Tissue culture**

Mammalian cells were grown at 37^o^ C with 5% CO_2_. THP-1 cells were grown in Roswell Park Memorial Institute (RPMI) 1640 media (Thermo Fisher Scientific) supplemented with 10% heat inactivated fetal bovine serum (FBS) (Thermo Fisher Scientific). Phorbol 12-myristate 13-acetate (PMA) (Sigma-Aldrich) at a final concentration of 20 ng/mL was used to differentiate THP-1 cells for 24 hrs. After this incubation, PMA-containing media was replaced with RPMI without PMA. A549 cells were grown in Dulbecco′s Modified Eagle′s Medium (DMEM) - high glucose (Sigma-Aldrich) supplemented with 10% heat inactivated FBS (Thermo Fisher Scientific). Seeded A549 cells were incubated for 24hrs before infection. Bone-marrow derived macrophages (BMDMs) were isolated from femurs of C57BL/6 mice by flushing with complete DMEM (DMEM supplemented with 10% fetal bovine serum, 5mM non-essential amino acids, and 5mM L-glutamine) (4). Cells were washed, resuspended, and plated in complete DMEM supplemented with 20% L-929 conditioned media (LCM) and incubated for 6 days. Macrophages were then harvested using cold 5mM EDTA in phosphate buffered saline (PBS) and seeded in complete DMEM supplemented with 10% LCM.

**Quantitation of phage after SYBR Gold staining**

In order to determine if the phages used are inactivated by SYBR Gold staining, phage were stained with SYBR Gold or incubated with equivalent phage buffer for 1 hr at 4^o^ C. Plaque assays were used to determine phage titer.

**Fluorescence microscopy of phage uptake by mammalian cells**

Cells were stained with 5 μg/mL CellMask deep red plasma membrane stain (Thermo Fisher Scientific) in PBS at 37^o^C for 5 minutes. The CellMask stain was then removed and cells were fixed with 4% paraformaldehyde (PFA) for 20 minutes at 25^o^C. Cells were then washed 3 times with PBS and stained with 100 ng/mL diamidino-2-phenylindole (DAPI) for five minutes at 25^o^C. Excess DAPI was removed by washing three times with PBS.

**Transmission electron microscopy**

Fixed cells were further processed by three rinses with 0.15 M sodium phosphate buffer, pH 7.4. Then the cells were post-fixed with 1% osmium tetroxide/1.25% potassium ferrocyanide/0.15M sodium phosphate buffer for 1 hour at RT (5). After washing in deionized water (three times), the cells were dehydrated using increasing concentrations of ethanol (30%, 50%, 75%, 100%, 100%, 10 minutes each) and embedded in Polybed 812 epoxy resin (Polysciences, Inc., Warrington, PA). The cells were sectioned *en face* to the substrate at 75 nm using a diamond knife and Leica Ultracut UCT ultramicrotome (Leica Microsystems, Inc., Buffalo Grove, IL). Ultrathin sections were collected on 200 mesh copper grids and stained with 4% aqueous uranyl acetate for 12 minutes, followed by Reynolds’ lead citrate for eight minutes (6). Samples were observed with a JEOL JEM-1230 transmission electron microscope operating at 80kV (JEOL USA, Peabody, MA) and digital images acquired using a Gatan Orius SC1000 CCD camera and Gatan Microscopy Suite 3.0 software (Gatan, Inc., Pleasanton, CA).

**Quantitation of phage and *M. abscessus* after PIB and mammalian cell lysis conditions**

In order to determine if the conditions used to lyse mammalian cells affected phage and *M. abscessus* titer, both phage and bacteria were incubated with 0.1% Triton X-100 +/- phage inactivation buffer (PIB; 40 mM citric acid, 10 mM KCl, 135 mM NaCl, pH 3.0) for 10 min at room temperature. The Triton X-100 and phage inactivation buffer treated sample was diluted, plated for CFU on 7H10 plates. To measure phage titer, the remaining sample was centrifuged for 5 min at 15,000 x g and the resulting supernatant was diluted and spotted on *M. smegmatis* top agar plates.

**References**

1. Freeman KG, Robotham AC, Parks OB, Abad L, Jacobs-Sera D, Lauer MJ, Podgorski JM, Zhang Y, Williams JV, White SJ, Kelly JF, Hatfull GF, Pope WH. 2023. Virion glycosylation influences mycobacteriophage immune recognition. Cell Host & Microbe 31:1216-1231.e6.

2. Bonilla N, Rojas MI, Netto Flores Cruz G, Hung SH, Rohwer F, Barr JJ. 2016. Phage on tap-a quick and efficient protocol for the preparation of bacteriophage laboratory stocks. PeerJ 4:e2261.

3. Goude R, Parish T. 2009. Electroporation of Mycobacteria, p 203-215. *In* Parish T, Brown AC (ed), Mycobacteria Protocols: Second Edition. Humana Press, Totowa, NJ.

4. Zulauf KE, Sullivan JT, Braunstein M. 2018. The SecA2 pathway of Mycobacterium tuberculosis exports effectors that work in concert to arrest phagosome and autophagosome maturation. PLoS pathogens 14:e1007011.

5. Russell L, Burguet S. 1977. Ultrastructure of Leydig cells as revealed by secondary tissue treatment with a ferrocyanide-osmium mixture. Tissue and cell 9:751-766.

6. Reynolds ES. 1963. The use of lead citrate at high pH as an electron-opaque stain in electron microscopy. The Journal of cell biology 17:208.
